# Healthy Food Prices Increased More Than the Prices of Unhealthy Options during the COVID-19 Pandemic and Concurrent Challenges to the Food System

**DOI:** 10.3390/ijerph20043146

**Published:** 2023-02-10

**Authors:** Meron Lewis, Lisa-Maree Herron, Mark D. Chatfield, Ru Chyi Tan, Alana Dale, Stephen Nash, Amanda J. Lee

**Affiliations:** School of Public Health, Faculty of Medicine, The University of Queensland, Herston 4006, Australia

**Keywords:** diet cost, diet affordability, food security, COVID-19, Australia

## Abstract

Food prices have escalated due to impacts of the COVID-19 pandemic on global food systems, and other regional shocks and stressors including climate change and war. Few studies have applied a health lens to identify the most affected foods. This study aimed to assess costs and affordability of habitual (unhealthy) diets and recommended (healthy, equitable and more sustainable) diets and their components in Greater Brisbane, Queensland, Australia from 2019 to 2022 using the Healthy Diets Australian Standardised Affordability and Pricing protocol. Affordability was determined for reference households at three levels of income: median, minimum wage, and welfare-dependent. The recommended diet cost increased 17.9%; mostly in the last year when the prices of healthy foods, such as fruit, vegetables and legumes, healthy fats/oils, grains, and meats/alternatives, increased by 12.8%. In contrast, the cost of the unhealthy foods and drinks in the habitual diet ‘only’ increased 9.0% from 2019 to 2022, and 7.0% from 2021 to 2022. An exception was the cost of unhealthy take-away foods which increased by 14.7% over 2019–2022. With government COVID-19-related payments, for the first time recommended diets were affordable for all and food security and diets improved in 2020. However, the special payments were withdrawn in 2021, and recommended diets became 11.5% less affordable. Permanently increasing welfare support and providing an adequate minimum wage, while keeping basic, healthy foods GST-free and increasing GST to 20% on unhealthy foods, would improve food security and diet-related health inequities. Development of a Consumer Price Index specifically for healthy food would help highlight health risks during economic downturns.

## 1. Introduction

Poor diet is the leading single preventable risk factor contributing to the burden of disease in Australia and globally [1]. Less than 4% of Australian adults consume a diet consistent with the recommendations of the Australian Dietary Guidelines (ADGs) [2], with over 35% of dietary energy derived from unhealthy discretionary food and drinks [3]. Relatedly, more than two thirds (67%) of Australian adults and 25% of children aged two to 17 years are overweight or obese [4]. In Australia, rates of obesity, poor-quality diet, and diet-related chronic disease (including type 2 diabetes, heart disease, and some cancers [1,5]) follow a socioeconomic gradient [6,7,8].

Ensuring food security is key to reducing prevalence of obesity and chronic disease particularly in low socioeconomic groups. Food security is a fundamental human right [9]. It means everyone is able to obtain at all times a sufficient quantity of quality (safe and nutritious) food that meets their preferences to sustain “an active and healthy life” [10]. Food security is determined by availability, accessibility, affordability, and acceptability of food.

The affordability of healthy diets is impacted by both the cost of food and drinks and the household financial resources available. Economic access to food implies that people “have sufficient money to purchase the food they want to eat, to meet cultural and social as well as health and nutritional norms; that this money is not absorbed in other expenditure demands (rent, fuel, debt repayment, etc.); [and] that people can … obtain food in ways which are dignified and in keeping with social norms” [11]. Income level, income shocks and rising costs of living are key determinants of economic access to healthy food [12,13,14].

As with all diets, recommended diets (which are healthy, equitable, and sustainable consistent with the Australian Dietary Guidelines (ADGs) [2]) are considered unaffordable when they cost more than 30% of household income [15]. When a household needs to spend more than 25% of their disposable income on healthy food, they may experience “food stress” and are vulnerable to food insecurity [16]. Before the COVID-19 pandemic, families relying on government welfare/financial assistance had to spend over a third of their household income to buy a recommended diet, and households in rural and remote areas on low incomes needed to spend an even greater proportion [17].

Prior to COVID-19, estimates of the prevalence of food insecurity in Australia were around 4 to 14% in the general population and up to 82% in low-income groups [18]. Over the past three years, food insecurity has increased significantly [19,20]. According to the latest national Hunger Report from Australian food charity Foodbank, extrapolating responses from a nationally representative survey conducted in July 2022, 21% of Australians had experienced severe food insecurity in the past 12 months (up from 17% in 2021) [19]. Of those experiencing food insecurity, 64% cited increased or high living expenses and 42% reported “reduced or low income or government benefits” as a key cause [19].

Previous studies have assessed diet costs during the COVID-19 pandemic and, opportunistically, the impact on diet affordability of government economic responses that boosted incomes for many low-income households [17,21]. In response to COVID-19 impacts, including increased unemployment, in 2020 the Australian Government introduced “JobKeeper” payments to help businesses pay employees who were stood down, and also lump sum Economic Support Payments and a fortnightly Coronavirus Supplement (hereafter abbreviated as ESP and CS) for eligible recipients of some income support payments, including the unemployment benefit “JobSeeker” [22]. Diet costs increased from 2019 to 2020, largely driven by rising prices of most healthy food groups [21]. However, increased income support meant that for the first time, welfare-dependent families had economic access to recommended diets [17,21].

Food prices have continued to escalate since 2020 due to global, national, and regional shocks and stressors, including the impacts of climate change, increasing frequency and severity of extreme weather events (bushfires and floods), the Russian invasion of Ukraine, and changing demographics (such as reduced immigration and increased internal migration to rural areas [23]), as well as disruptions to food production and supply due to impacts on workforces of COVID-19-related public health restrictions [24,25,26,27]. These factors also contributed to increased costs of fuel, feed, and fertiliser, exacerbating increasing food prices.

This study aimed to assess costs of habitual (unhealthy) diets and recommended diets in Greater Brisbane in 2021 and 2022, and compared results with those reported from earlier studies in 2019 and 2020 [21], to explore changing costs and affordability in the context of these shocks and stressors.

## 2. Materials and Methods

The Healthy Diets ASAP (Australian Standardised Affordability and Pricing) methods protocol [28] was applied to assess the cost, cost differential, and affordability of habitual (unhealthy) and recommended (healthy, more equitable and sustainable) diets in the Greater Brisbane region of Queensland, Australia annually from 2019 to 2022. Findings from 2019 and 2020 have been previously reported [17,21]. This study collected and analysed data in 2021 and 2022 and compared diet cost, cost differentials, and affordability across the four timepoints. All cost values are provided in Australian dollars ($).

The Healthy Diets ASAP protocol is consistent with the International Network for Food and Obesity/non-communicable diseases Research, Monitoring and Action Support (INFORMAS) framework’s ‘optimal’ approach to assess diet price and affordability [29], addressing limitations of earlier efforts to measure food cost and affordability in Australia [30,31]. Details of the background, description, collaborative development process, application, and testing of the protocol have been published previously [28,32].

The protocol has five parts: standardised habitual and recommended diet pricing tools; store location and sampling; calculation of median gross and minimum wage disposable income; food price data collection; and analysis and reporting [28].

### 2.1. Diet Pricing Tools

The diet pricing tools specify the types of foods and drinks in the habitual and recommended diets and quantities for a reference household of four (adult male and female 31–50 years of age, a 14-year-old boy, and an 8-year-old girl) per fortnight [28]. The recommended diet contains healthy food and drinks, in line with the recommendations of the ADGs [2]. The habitual diet is based on reported dietary intake data from the most recent Australian Health Survey National Nutrition and Physical Activity Survey (NNPAS) 2011–2013 [3]. It includes some healthy food and drinks in lower amounts than recommended in the ADGs, and many discretionary foods and drinks (defined by the ADGs as not being necessary for health and high in saturated fat, added sugar, sodium and/or alcohol) [2]. The recommended diet contains slightly less energy (33,610 kJ/day) for the reference household than the habitual diet (33,869 kJ/day), and is more sustainable, requiring less water, protecting biodiversity, and generating 25% lower greenhouse gas emissions in its production [33]. Table 1 lists the types of food and drinks included in each diet pricing tool. Detailed lists of components and quantities have been published previously [28].

### 2.2. Store Locations and Sampling

Food and drink price data for 2019 were sourced from a previous survey using the Healthy Diets ASAP protocol to assess diet costs and affordability in locations throughout Queensland [17,21]. The Australian Bureau of Statistics’ (ABS) Statistical Geography Standard classes medium-sized geographical areas into SA2 locations, where communities “interact together socially and economically” [34]. In 2019, SA2 locations across Queensland were stratified into quintiles of socioeconomic disadvantage based on the Socioeconomic Indexes for Areas (SEIFA) Index of Relative Socioeconomic Disadvantage [35]. Eighteen locations in SEIFA quintiles 1 (most disadvantaged), 3 (median disadvantaged) and 5 (least disadvantaged) were randomly selected for inclusion; the final samples included 10 locations in Greater Brisbane (3, 4, and 3 locations in SEIFA quintiles 1, 3, and 5, respectively). In 2020, restriction of movement implemented as a public health measure in response to the COVID-19 pandemic meant data collection in stores beyond Greater Brisbane was not possible. Hence, only the 10 SA2 locations in Greater Brisbane included in the 2019 sample were re-surveyed for food prices in 2020 [21]. Additionally, because of the pandemic, one of the large supermarket chains did not allow ‘unnecessary’ store visits (e.g., for research purposes) so prices from that supermarket were collected from its website matched to the SA2 locations. Previous studies comparing in-store to online prices have found insignificant price differences [36].

In similar months in 2021 and 2022, food prices were collected in the same 10 locations in Greater Brisbane. At each location, two large supermarkets (one of each major supermarket chain), an independent grocery store, a bakery, a fish and chip shop, two fast food restaurants, and one alcohol outlet were surveyed. As per the Healthy Diets ASAP protocol [28], if a store had closed since the previous survey, a similar, proximate food outlet was surveyed instead. In 2021 and 2022, prices were collected online for the two supermarkets in all locations and collected in-store for the remaining outlets. In 2022, a duplicate set of prices were collected in-store from the large supermarkets in two locations for validation.

### 2.3. Price Data Collection

Price data were collected by trained research assistants, following the Healthy Diets ASAP protocol, between August and October each year. Collection of price data in 2019 and 2020 has been detailed elsewhere [17,21]. In 2021, food price data were collected by L.-M.H. and R.C.T. and in 2022 by L.-M.H., M.L., A.D., and S.N. Permission to collect data was requested and received from national head offices of large supermarket chains, and also from store managers in each outlet. The data collection protocol outlines the procedure followed if the stipulated brands and sizes were not available or were on price promotion [28].

### 2.4. Household Income Calculation

Household incomes were calculated as per the Healthy Diets ASAP protocol [28], using publicly available national data from government agencies. This study assessed diet affordability for three categories of income for the reference household.

For each timepoint, the median gross household income (before taxation) per fortnight in each SA2 area was sourced from the ABS 2016 Census Community Profile [37] and adjusted by the ABS Wage Price Index [38]. Minimum wage disposable and welfare-dependent household incomes were calculated based on the set of assumptions detailed in the protocol, using payment entitlement data from Services Australia [39]. Calculations of minimum wage and welfare-dependent household incomes for 2020 included the ESP and CS provided between May and September 2020, are detailed elsewhere [21].

### 2.5. Analysis and Reporting

Food and drink price data were entered into the Healthy Diets ASAP data collection web portal [40] by R.C.T., A.D. and S.N. Data were double entered, and any discrepancies were resolved by consensus. Data were cleaned and checked by M.L. and A.J.L. As per the Healthy Diets ASAP protocol, if a value was missing, the mean price of the item in other stores in the same SA2 location was substituted. Spreadsheet algorithms generated results for each location in Microsoft Office Excel files which were cross-checked by M.L. and A.J.L.

Diet costs and affordability were calculated for each SA2 area surveyed in Greater Brisbane. The mean costs of the habitual and recommended diets, and the cost and proportion of the total spent on different ADG food groups and components, were calculated for the reference household per fortnight. Results were reported for SA2 SEIFA quintile 1, 3, and 5, and for Greater Brisbane as a whole. Affordability of habitual and recommended diets was calculated for households with the three different income levels described above.

The results for each year were compared to relevant findings of previous surveys, to assess changes in diet costs, cost of ADG food groups and components, and affordability of the diets. Consumer Price Index of food and non-alcoholic beverages (CPI-food) data for Brisbane from 2019 to 2022 were sourced from the ABS [41] for comparison with observed changes in food prices. Statistical analysis was conducted by paired *t*-tests; statistical significance was set at *p* ≤ 0.05.

## 3. Results

### 3.1. Selected Locations and Stores Surveyed

In 2019, food prices were collected from 80 outlets in 10 locations in Greater Brisbane [17]; in 2020, price data were collected in-store from 68 outlets and online from 10 outlets (supermarkets) [21]; in 2021 prices were collected from 80 outlets: in-store for 60 and online for 20 (supermarkets); and in 2022, prices were collected from 80 outlets: in-store for 60 and online for 20 (supermarkets). For validation, prices were re-collected in-store for four supermarket outlets across two locations.

### 3.2. Diet Cost Data

**Figure 1:**
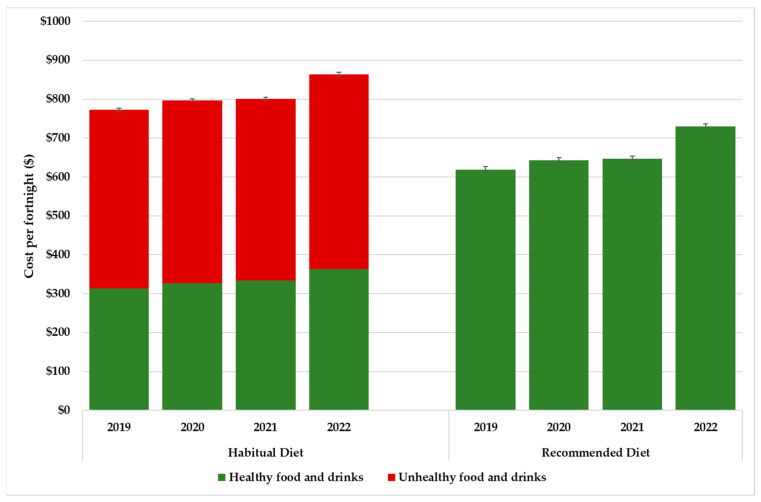
Cost of habitual and recommended diets per fortnight for the reference household of four, Greater Brisbane, 2019 to 2022.

**Figure 2:**
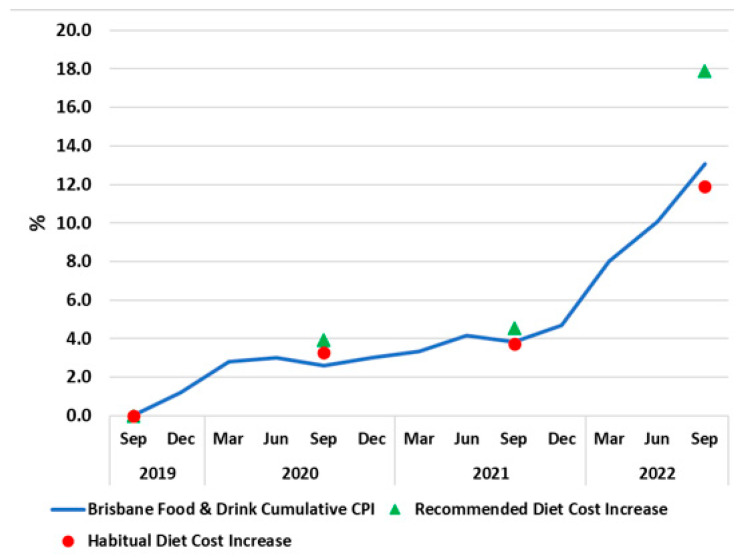
Cumulative increase in the costs of recommended and habitual diets and Consumer Price Index (CPI) increases, Brisbane, 2019 to 2022.

The mean costs ± standard error (SE) of the habitual and recommended diets and diet components in Greater Brisbane from 2019 to 2022 are reported in Table 2. Total costs of the recommended diets and the healthy and discretionary (unhealthy) components of the habitual diets for the four timepoints are also presented in Figure 1. Diet cost data for each location surveyed (Supplementary Figures S1 and S2) and by SEIFA quintile (Supplementary Table S1) are presented in supplementary files. Cumulative increase in the cost of the habitual and recommended diets and CPI-food is shown in Figure 2.

Within the manuscript, data are presented and analysed for Greater Brisbane as a whole, as change in food prices was similar regardless of SEIFA quintiles (Supplementary Table S1). The duplicated diet costs for prices collected in-store were within 1% of diet costs for prices collected online from the large supermarkets in two locations.

### 3.3. Diet Cost in Greater Brisbane, 2022

In 2022, the mean cost of the recommended diet in Greater Brisbane was AUD 729.71 per fortnight for the reference household of two adults and two children (Table 2). The habitual diet was 18.4% more expensive than the recommended (healthy) diet at a cost of AUD 863.93 (Table 2). Shifting from a habitual to recommended diet would save households AUD 134.22 per fortnight on average.

In 2022, nearly 58% of the total cost of the habitual diet was required to purchase discretionary food and drinks, including takeaway foods (around 20% of total diet cost), alcoholic drinks (11% of total diet cost), and sugar sweetened drinks (around 4% of total diet cost) (Table 2).

### 3.4. Changes in Fortnightly Diet Costs over Time

Between 2019 and 2022, in Greater Brisbane the cost of the recommended diet increased by 17.9% from AUD 619.04 to AUD 729.71 (*p* < 0.001) (Table 2). Around three-quarters of this increase occurred in the 12 months prior to the most recent survey in 2022, during which time the cost of healthy foods increased 12.8% (*p* < 0.001) (Table 2, Figure 1).

Over the four timepoints, the cost of the habitual diet increased 11.9% from AUD 772.20 to AUD 863.83 (*p* < 0.001). Again, most of the rise occurred between 2021 and 2022, during which period the total cost of the habitual diet increased by 7.8% (*p* < 0.001). This was around half the rate of increase for the recommended diet; the main reason for this was that the cost of the discretionary (unhealthy) food and drinks in the habitual diet only increased by 7.0% (*p* < 0.001) during that year (Table 2; Figure 1).

The CPI-food for the period September quarter 2021 to September quarter 2022 in Brisbane was 8.6% [41], so the increase in the cost of the recommended diet (12.8%) was 49% higher than the relevant reported CPI-food and even higher (64%) than the increase in cost of the habitual diet (7.8%), as illustrated in Figure 2. For the two-year period prior to September 2021, the CPI-food for Brisbane increased by 3.8%. During this period, the assessed cost increases of the recommended diet and the habitual diet were much closer to CPI-food; however, the cost increases in the recommended diet tended to be higher than those in the habitual (unhealthy) diet. This differential was exacerbated in 2022 (Figure 2).

The differential between the cost of the habitual and recommended diets was 24.7% in 2019 and was similar for the following two years, being 23.9% in 2020 (*p* = 0.90) and 23.8% in 2021 (*p* = 0.98). However, the cost differential declined significantly to 18.4% from 2021 to 2022 (*p* < 0.001). In 2022, the recommended diet was relatively more expensive than it had been compared to the habitual diet in 2019 (*p*= 0.006).

### 3.5. Changes in Fortnightly Cost of Food Groups over Time

In the recommended diet, food groups with the highest cost increases from 2019 to 2022 and from 2021 to 2022, respectively, were: vegetables and legumes (20.5% and 34.9%, both *p* < 0.001), grain (cereal—mostly wholegrain) foods (15.6% and 11.4%, both *p* < 0.001), fruit (23.4%, *p* < 0.001 and 8.2%, *p* = 0.010), and healthy fats and oils (27.3%, 17.5%, both *p* < 0.001) (Table 2). Notably the cost of ‘vegetables and legumes’ had decreased from 2019 to 2020 (−12.7%, *p* < 0.001) before increasing from 2020 to 2021 (4.8%, *p* < 0.001) and then increased markedly from 2021 to 2022 (34.9%, *p* < 0.001), as noted previously. Costs of products in the milk, cheese, and yoghurt group increased initially from 2019 to 2020 (7.4%, *p* = 0.012), before declining slightly from 2020 to 2021 (−3.1%, *p* = 0.018), then rising most significantly from 2021 to 2022 (10.3%, *p* < 0.001); an overall increase of 14.7% (*p* < 0.001) from 2019 to 2022. The cost increases in the lean meats, poultry, fish, eggs, and plant-based alternatives group were more consistent throughout the three years, increasing by 6.1% from 2019 to 2020 (*p* = 0.011), 3.5% from 2020 to 2021 (*p* = 0.013), and 6.4% from 2021 to 2022 (*p* < 0.001), leading to an overall increase of 16.9% (*p* < 0.001) from 2019 to 2022.

From 2019 to 2022, in contrast to the marked increase in the cost of the healthy foods and drinks in the recommended diet (17.9%), the cost of all the discretionary (unhealthy) foods and drinks in the habitual diet increased by ‘only’ 9.0% (Table 2). There was also a large variation in the price changes within individual components of the latter. For example, the price of alcoholic drinks was relatively stable from 2019 to 2020, decreased in the first years of the COVID-19 pandemic (−6.4%, *p* < 0.001), then increased by 3.2% from 2021 to 2022 (*p* < 0.001), leading to an overall decrease of −1.8% (*p* < 0.001) from 2019 to 2022. The cost of sugar sweetened beverages followed a similar pattern, increasing by 12.3% (*p* < 0.001) from 2021–2022, while the cost of artificially sweetened beverages increased throughout the four timepoints by 21.5% (*p* < 0.001) (Table 2). Among discretionary (unhealthy) items, the highest price increases from 2019 to 2022 were in take-away foods (14.7%, *p* < 0.001); while increasing by 5.7% (*p* < 0.001) from 2019 to 2020, the cost of takeaway foods did not change significantly in the first year of the COVID-19 pandemic, then increased from 2021 to 2022 (6.0%, *p* < 0.001).

### 3.6. Changes in Diet Affordability over Time

**Figure 3:**
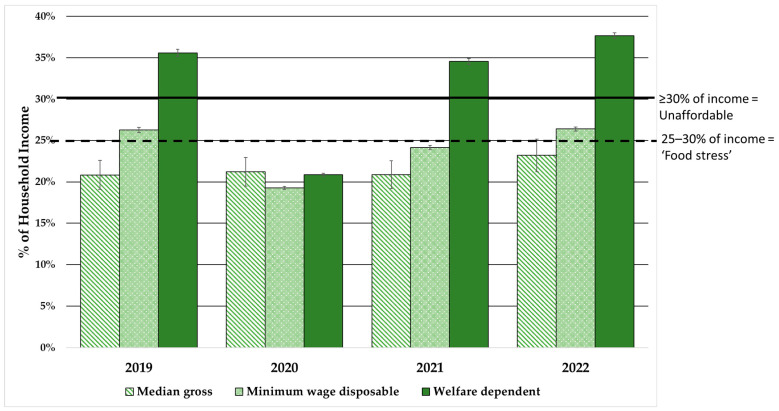
Affordability of the recommended diet for reference household of four in Greater Brisbane, 2019 to 2022.

Table 3 presents household incomes at the three different levels for the reference household per fortnight and affordability of the recommended diet in Greater Brisbane from 2019 to 2022. Affordability of the recommended diet over time is also presented in Figure 3. Calculations of the minimum wage disposable household incomes and welfare-dependent household incomes at the four timepoints are provided in Supplementary Tables S2 and S3.

In 2019, median gross household income per fortnight (AUD 3188.00) was 35% higher than household income for those on minimum wage (AUD 2358.00) and 83% higher than households on welfare income (AUD 1739.68). Due to the ESP and CS payments, both the minimum wage disposable household income and welfare-dependent household income in Greater Brisbane increased markedly between May and September 2020, by 41.5% and 77.3%, respectively. After the ESP and CS payments ceased early in 2021, both incomes returned to levels only 17% and 11% higher, respectively, than in 2019 (Table 3).

In 2019, the reference household on median household income would have needed to spend 20.8% of their income to purchase the recommended diet in Greater Brisbane, which was affordable. Those households on minimum wage had to pay 26.0% of their household income to purchase the recommended diet, so would have been in food stress. However, welfare-dependent households could not afford the recommended diet, which cost 35.6% of household income (Table 3, Figure 3).

In contrast, for the first time, due to the ESP and CS introduced in 2020, the recommended diet was affordable for the reference household on welfare income, costing 20.3% of household income (Table 3, Figure 3). In addition, in 2020 the household on minimum wage was no longer in food stress, with the recommended diet costing 19.3% of household income. Hence, affordability of the recommended diet and economic access to healthy diets was similar for vulnerable households and those on median income in 2020. However, the ESP and CS were withdrawn incrementally from late 2020 and removed in early 2021. In 2021, welfare-dependent households again could not afford recommended diets, which cost 34.6% of household income. Those on minimum wage fared better at this time, narrowly avoiding food stress as the recommended diet cost 24.1% of their household income. However, diet affordability continued to worsen in all households with the increasing cost of food from 2021 to 2022. While approaching the food stress threshold, those on median income could still afford the recommended diet which cost 23.2% of their household income in 2022. Those on minimum wage were again experiencing food stress with the recommended diet costing 26.4% of their household income. Most worryingly, recommended diets were once again unaffordable for welfare-dependent households, costing 37.7% of their household income. Since 2019, economic access to healthy diets had reduced by 11.5% for households on median income, 1.5% for those on minimum income, and 5.0% for those on welfare.

## 4. Discussion

The Healthy Diets ASAP protocol was applied to assess the cost, relative cost, and affordability of habitual (unhealthy) diets and recommended (healthy, equitable and sustainable) diets, for a reference family of two adults and two children at three different household income levels in Greater Brisbane once a year from 2019 to 2022. This enabled comparison of diet costs and affordability at timepoints before and during the COVID-19 pandemic and concurrent shocks and stresses at global, national, and regional levels that may have impacted economic aspects of food security in Greater Brisbane.

The study found that from 2019 to 2021 prices of food and drinks in greater Brisbane increased gradually consistent with the CPI-food of 3.8% but escalated markedly between 2021 and 2022 (Figure 2). Between September 2021 and 2022, the cost of the healthy food and drinks comprising the recommended diet increased by an average of 12.8%, nearly double the rate of the increase in the cost of the discretionary foods and drinks in the habitual diet during the same period (7.0%), and 42% more than the Brisbane CPI-food in that period (8.6%).

A major contributor to the increased cost of a healthy diet from 2021 to 2022 was the increase in fruit, vegetable, and grain prices that has been attributed to heavy rainfall and flooding in key Queensland food production areas during that year [42]. Other previous natural disasters, including the wild bushfires of 2019–2020, were likely to have ongoing impacts on supply [21,43]. In October 2022, the Australian Government Treasury predicted fruit and vegetables prices would increase a further 8% in the six months following due to new flood events in Australian food growing regions [42].

Continuing COVID-19-related supply chain disruptions were also reported. These included reduced workforce, such as reduced availability of international seasonal fruit pickers due to Australian border closures and forced isolation of workers in manufacturing, distributing, transport, and retail, including those with COVID-19 and close contacts [44,45]. Other factors included high fuel, and hence high transport costs, and high fertiliser and feed costs due to the Russian invasion of Ukraine [25,27]. The war has also been linked with increased cost of cereal products/bread due to constrained global wheat supply and increased cost of cooking oil [26,46], which grew by 17.5% in the last year of this study.

Another key factor was increasing global and national inflation [25,41]. In Australia, this exacerbated the increased price of gas and electricity, which are used for cooking, and of fuel used for shopping [41]. The escalating cost of living also pressured essential spending, such as on rent and mortgages for housing, which impacted spending on food [41]. As cash rates rise to combat inflation, this can lead to increased interest payments on borrowing, also increasing pressure on household budgets [41].

As prices of healthy foods escalate, unhealthy (discretionary and/or ultra-processed) foods have become relatively cheaper, which influences dietary choices, particularly in an economic downturn [47,48]. In times of financial stress, families, particularly in low socio-economic groups, tend to purchase the cheapest and most affordable food; this is often the less healthy products on price promotion [47,48]. This is worrying given that, when last measured nationally in Australia (2011–2012), more than one third of adults’ energy intake, and nearly 40% of children’s, was derived from unhealthy, discretionary food [49]. Total intake of discretionary food and drinks is associated with increased body mass index, and lower consumption of fruit and vegetables [50]. The relatively greater increase in the price of healthy, compared to discretionary, food and drinks during the study likely contributed to reported reductions in the intake of fresh produce, increased intake of unhealthy foods and weight gain during the COVID-19 pandemic [51,52].

Price is just one of many factors influencing dietary choices. Other aspects of the ‘obesogenic’ food environment [53] that drive food choices include the constant availability and promotion of unhealthy food and drinks, and convenience. These factors may explain why the cost of take-away foods increased more than other unhealthy foods and drinks during this study (Table 2). Better promotion of healthy food and drinks and improvement in the nutrient profile of some convenience foods (e.g., reduction of salt content [54]) may contribute to healthier dietary intakes.

A common perception that healthy foods are more expensive than unhealthy options is also relevant [2,55,56,57,58]. However, the studies supporting this are not grounded in reported dietary intakes such as the Healthy Diets ASAP protocol [28,29,32]. For example, they often cost arbitrary ‘healthy’ and ‘unhealthy’ food lists and often exclude alcohol and take-away foods, which comprise 20–25% of the cost of habitual Australian diets [28]. Results are also reported in different units (price per energy or weight unit, serve or nutrient-density) and analysis is frequently spurious [59]. Corresponding with findings of previous research, this study confirmed that healthy diets can be less expensive than habitual diets. Studies applying the Healthy Diets ASAP protocol have found that habitual diets are 14% to 23% more expensive than the recommended diet in Brisbane [28,32], across regional and remote areas of Queensland [17], in Sydney and Canberra [60], in regional Victoria [61], in remote Aboriginal and Torres Strait Islander communities [62,63], and nationally in areas serviced by the two large supermarket chains [64].

However, the cost differential between the diets reduced in 2022, reflecting the differing pricing and relative composition of the habitual and recommended diets. As the recommended diet comprises only healthy food and drinks, its total cost increased at a higher rate than the total cost of the habitual diet, which includes lesser quantities of healthy foods but many unhealthy items. A similar differential decrease was identified also in remote Aboriginal communities in Central Australia; in these communities between May 2021 and June 2022, the cost of the habitual diet increased approximately 5%, while the cost of the recommended diet increased by around 10% [65].

Despite the reduced cost differential (18.4% in 2022 down from 24.7% in 2019), the cost saving for households buying the recommended diet rather than the habitual diet remained significant in 2022—AUD 134.22 per fortnight for the reference household in Greater Brisbane. However, this was approximately AUD 20 per fortnight less than during the preceding years. Especially at a time of increased pressure on household budgets and financial stress [66], this likely reduced the financial incentive for households to improve their diet. Urgent action is needed to further increase the relative affordability of recommended diets compared to habitual diets.

The lower cost of recommended diets compared to habitual diets is largely attributable to the exemption of “basic, healthy foods” from the 10% Goods and Services Tax (GST) in Australia, which helps to keep the relative price of healthy food and drinks down. Modelling studies support increasing the GST on unhealthy food and drinks to 20% to increase the incentive for consumers to choose healthier options [32,67]. Targeted levies, such as 30% GST on sugar-sweetened beverages also have been suggested to discourage intake of specific choices harmful to health [68].

Analysis of data collected annually from 2019 to 2022 highlighted the impact on affordability of recommended diets during the temporary increases to income support for the most vulnerable households during the early months of the COVID-19 pandemic. The ‘natural experiment’ created when the Australian Government provided the ESP and CS, effectively doubling welfare income for those on JobSeeker in 2020, demonstrated that increasing household income raised an estimated 646,000 people above the poverty line [69] and made healthy diets affordable for welfare-dependent families for the first time [21].

Affordability of the recommended diet improved dramatically for low-income households in 2020, due to government provision of the ESP and CS from April. Affordability of recommended diets improved by 42% for welfare-dependent households. More than 90% of recipients of the ESP and CS income supplements reported that they could afford more healthy foods, including fruit and vegetables [70].

Findings of other studies and surveys confirm a positive impact of increased income support on food security and households’ capacity to buy healthy food [19,71] and subsequent increases in the prevalence of poverty and food insecurity after the income supplements decreased and then ceased (March 2021) [19,72]. Recommended diets became unaffordable for welfare-dependent households again, requiring 34.6% of their disposable income in 2021, and 37.7% in 2022. Families unable to commit such a large proportion of their disposable income to food because of other escalating living costs (particularly rent, household utilities such as electricity, and fuel/transport) report not being able to buy enough food or skipping meals to save money [73]. Low household income is the most consistent determinant of food insecurity [74].

The results of this study have highlighted that CPI-food is a blunt economic instrument. To help more usefully and transparently identify the likely health consequences of the changing prices of foods, it is recommended that the CPI-healthy food be determined and reported. A potential model for this was the ABS Australian Dietary Guidelines CPI reported in 2015 [75].

### Limitations

For this study it would have been ideal if COVID-19-related ‘JobKeeper’ payments made to businesses to support staff whose working hours were reduced during the pandemic could have been included in addition to ‘JobSeeker’ payments. However, it was too difficult to tease out ‘JobKeeper’ entitlements and payments at the household level; hence, the incomes calculated only reflect those made primarily to low-income households. It should be noted that median income may have varied for some employees ‘stood down’ during the early years of the COVID-19 pandemic. However, this would not have affected the findings relevant to households on minimum wage or dependent on welfare.

There are inherent methodological limitations to the Healthy Diets ASAP protocol that have been reported elsewhere [28]. Most pertinent to this analysis, they include assumptions used in income calculations that did not include the possibility of low-income households receiving the JobKeeper supplement in 2020, for example.

The most recent available national dietary intake data were collected in Australia in 2011–2012 [3]. It is likely ‘habitual’ diets of Australian families have changed in line with changes in the food supply and environment in recent years, including the rapid growth of meal delivery services [76,77], but recent granular data on diet patterns are not available currently. It is anticipated that updated national dietary intake data will be available in 2025 from the planned 2023 Australian Intergenerational Health and Mental Health Study (incorporating the National Nutrition and Physical Activity Survey) [78]. For the first time, national food security data will also be assessed robustly in this study [78].

The price collection protocol of Healthy Diets ASAP includes collection of the prices of major Australian brands for packaged food and drinks. Some households, particularly low socioeconomic households, may choose to purchase lower priced generic products (‘home’ or ‘own’ brands) and/or shop at ‘budget’ supermarkets as a coping strategy to stretch food budgets [79]. However, when applying this strategy, recommended diets can still be stressful to afford for welfare-dependent households [79], and are likely to be more so given the identified price rises of unpackaged healthy foods (such as fruit and vegetables) in this study.

Surveys were conducted only in urban locations in one state capital city in Australia, hence observed changes in food costs are not generalisable directly to other locations nationally, or other countries internationally. However, particularly for low-income groups, they do highlight current challenges in maintaining economic access to food security and healthy diets and help explain diet-related health inequities seen in Australia [79,80].

## 5. Conclusions

Having the ability to adequately feed ourselves is a basic human right; this requires that food must be affordable without compromising any other essential needs, such as secure housing (rent) or medicines [81]. Over the three years since 2019, the cost of food and drinks has escalated, particularly in the most recent year, with the prices of healthy foods and drinks increasing at almost double the rate of increase in prices of unhealthy items. The recommended diet remains less expensive than the habitual diet, but the cost differential reduced significantly between 2021 and 2022, rendering recommended diets relatively less affordable than habitual (unhealthy) diets. The development and publication of a CPI-healthy food index would help better identify health and related economic risks.

Findings highlight the potential impact of government policy settings on the affordability of food and economic aspects of food security, and hence dietary choices and diet-related health such as obesity, cardiovascular disease, type 2 diabetes, and some cancers. Despite effective relief conferred by the ESP and CS while paid, recommended diets have become increasingly unaffordable for Australian families on low income, and are less affordable now than before the COVID-19 pandemic. Affordability of healthy food could be guaranteed by government commitments to help families access essential needs, and also by commitments to keep basic, healthy food and drinks GST-free. Additional benefits would be delivered if GST on unhealthy foods and drinks was increased to 20%. This could help encourage healthier choices, and the revenue raised could be hypothecated to nutrition and health promotion programs. Permanently increasing welfare support and providing an adequate minimum wage would enable low-income households to meet the costs of living and afford adequate healthy food, to protect their food security and diet-related health.

## Figures and Tables

**Table 1:** Food and drinks included in the Healthy Diets ASAP diet pricing tools.

Habitual (Unhealthy) Diet	Recommended (Healthy, Equitable, More Sustainable) Diet
Healthy foods and drinks as per the seven food groups in the ‘Recommended diet’ column; in reduced amounts reflecting reported intakes Artificially sweetened beverages Discretionary (unhealthy) foods and drinks: ○ Drinks: sugar sweetened beverages ○ Cereals, snacks, and desserts: muffin, sweet biscuits, savoury crackers, confectionary, chocolate, potato crisps, muesli bar, mixed nuts (salted), ice cream, fruit salad (canned in juice) ○ Processed meats: beef sausages, ham ○ Spreads, sauces, condiments, and ingredients: butter, tomato sauce, salad dressing, white sugar ○ Convenience meals: frozen lasagne, chicken soup (canned), frozen fish fillet (crumbed), instant noodles, meat and vegetable stew (canned) ○ Fast food: pizza, meat pie, hamburger, potato chips/fries ○ Alcohol: beer (full strength), white wine (sparkling), red wine, whisky	Water (bottled) Fruit: apples, bananas, oranges Vegetables: potatoes, broccoli, white cabbage, iceberg lettuce, onion, carrot, pumpkin, tomatoes, sweetcorn (canned), four bean mix (canned), diced tomatoes (canned), baked beans (canned), frozen mixed vegetables, frozen peas, salad vegetables in sandwich Grain (cereals): wholegrain cereal biscuits (Weet-bix™), rolled oats, cornflakes, wholemeal bread, white bread, white rice, white pasta, dry water crackers, bread in sandwich Lean meats and alternatives: beef mince and steak, lamb chops, cooked chicken, tuna (canned), eggs, peanuts (unsalted), meat in sandwich Milk, yoghurt, and cheese: cheddar cheese (full fat, reduced fat), milk (full fat, reduced fat), yoghurt (full fat plain, reduced fat flavoured) Unsaturated oils and spreads: olive oil, sunflower oil, canola (margarine)

**Table 2:** Cost of habitual and recommended diets and component food groups for the reference household (two adults and two children) per fortnight in Greater Brisbane, in 2019, 2020, 2021 and 2022.

Total Diet and Food Group Costs of the Habitual Diet for the Reference Household								
	2019		2020		2021		2022	
Food/Food Groups	Mean Cost (AUD) ± SE	Proportion of Total Cost (%)	Mean Cost (AUD) ± SE	Proportion of Total Cost (%)	Mean Cost (AUD) ± SE	Proportion of Total Cost (%)	Mean Cost (AUD) ± SE	Proportion of Total Cost (%)
Water, bottled	20.35 ± 0.52	2.64%	19.04 ± 0.55	2.39%	19.62 ± 0.37	2.45%	20.27 ± 0.48	2.35%
Fruit	53.38 ± 1.29	6.91%	57.44 ± 0.72 *	7.20%	57.02 ± 0.96	7.12%	58.45 ± 1.01 ^^	6.77%
Vegetables (and legumes)	43.59 ± 0.59	5.65%	40.51 ± 0.53 **	5.08%	40.93 ± 0.52	5.11%	52.38 ± 0.58 *** ^^^	6.06%
Grain (cereal) foods	44.34 ± 0.63	5.74%	46.17 ± 0.52 *	5.79%	46.6 ± 0.69	5.82%	52.08 ± 0.54 *** ^^^	6.03%
Lean meats, poultry, fish, eggs, nuts, seeds and alternatives	96.45 ± 1.15	12.49%	101.86 ± 1.31 *	12.77%	107.12 ± 1.36 **	13.37%	112.51 ± 1.38 ** ^^^	13.02%
Milk, yoghurt, cheese and alternatives	47.93 ± 0.87	6.21%	55.02 ± 0.42 ***	6.90%	54.25 ± 0.38 *	6.77%	58.91 ± 0.66 *** ^^^	6.82%
Unsaturated oils and spreads	1.27 ± 0.02	0.17%	1.30 ± 0.02 *	0.16%	1.41 ± 0.02 ***	0.18%	1.68 ± 0.02 *** ^^^	0.19%
Artificially sweetened beverages	5.64 ± 0.14	0.73%	6.14 ± 0.07 **	0.77%	6.14 ± 0.07	0.77%	6.85 ± 0.05 *** ^^^	0.80%
Sugar sweetened beverages	31.14 ± 0.50	4.03%	30.82 ± 0.32	3.87%	30.86 ± 0.33	3.85%	34.66 ± 0.46 *** ^^^	4.01%
Takeaway foods	149.31 ± 2.22	19.34%	157.76 ± 2.34 ***	19.79%	161.59 ± 1.78	20.17%	171.29 ± 3.20 *** ^^^	19.83%
Alcoholic beverages	96.36 ± 1.91	12.48%	97.93 ± 0.91	12.28%	91.71 ± 0.27 ***	11.45%	94.62 ± 0.72 **	10.95%
All other discretionary choices	182.42 ± 3.01	23.62%	183.38 ± 2.09	23.00%	183.88 ± 1.52	22.95%	200.22 ± 2.08 *** ^^^	23.18%
**Total diet**	**772.20 ± 4.48**	**100.00%**	797.36 ± 3.79 ***	**100.00%**	**801.13 ± 3.99**	**100.00%**	863.93 ± 5.59 *** ^^^	**100.00%**
Healthy foods and drinks	312.96 ± 3.23	40.53%	327.47 ± 2.55 **	41.07%	333.09 ± 3.01 **	41.58%	363.14 ± 3.09 *** ^^^	42.03%
Discretionary foods and drinks	459.24 ± 2.38	59.47%	469.89 ± 2.90 *	58.93%	468.04 ± 1.49	58.42%	500.79 ± 3.75 *** ^^^	57.97%
**Total diet and food group costs of recommended diet for the reference household**								
	**2019**		**2020**		**2021**		**2022**	
**Food/Food groups**	**Mean cost (AUD) ± SE**	**Proportion of total cost (%)**	**Mean cost (AUD) ± SE**	**Proportion of total cost (%)**	**Mean cost (AUD) ± SE**	**Proportion of total cost (%)**	**Mean cost (AUD) ± SE**	**Proportion of total cost (%)**
Water, bottled	20.35 ± 0.52	3.29%	19.04 ± 0.55	2.96%	19.62 ± 0.37	3.03%	20.27 ± 0.48	2.78%
Fruit	72.81 ± 2.52	11.76%	88.31 ± 1.95 ***	13.72%	83.01 ± 1.89 ***	12.83%	89.82 ± 1.10 * ^^^	12.31%
Vegetables (and legumes)	110.36 ± 1.70	17.83%	96.97 ± 1.43 ***	15.07%	101.67 ± 1.5 ***	15.71%	137.14 ± 1.22 *** ^^^	18.79%
Grain (cereal) foods	109.99 ± 0.75	17.77%	113.86 ± 1.25 *	17.69%	113.98 ± 1.81	17.61%	126.95 ± 1.79 *** ^^^	17.40%
Lean meats, poultry, fish, eggs, nuts, seeds and alternatives	184.52 ± 2.76	29.81%	195.83 ± 2.39 *	30.43%	202.68 ± 2.22 *	31.32%	215.63 ± 3.61 ** ^^^	29.55%
Milk, yoghurt, cheese and alternatives	112.59 ± 2.64	18.19%	120.88 ± 1.69 *	18.79%	117.1 ± 1.17 *	18.09%	129.18 ± 1.83 *** ^^^	17.70%
Unsaturated oils and spreads	8.42 ± 0.12	1.36%	8.59 ± 0.12	1.33%	9.12 ± 0.15 **	1.41%	10.72 ± 0.14 *** ^^^	1.47%
**Total diet**	**619.04 ± 7.16**	**100.00%**	643.47 ± 5.84 *	**100.00%**	**647.18 ± 6.94**	**100.00%**	729.71 ± 6.30 *** ^^^	**100.00%**

* indicates *p* ≤ 0.05 difference from previous year; ** indicates *p* ≤ 0.01 difference from previous year; *** indicates *p* ≤ 0.001 difference from previous year; ^^ indicates *p* ≤ 0.01 difference from 2019; ^^^ indicates *p* ≤ 0.001 difference from 2019; **Bold type** indicates the total diet cost.

**Table 3:** Household incomes (for the reference household of four, per fortnight) and affordability * of the recommended diet, Greater Brisbane, 2019 to 2022.

	2019		2020		2021		2022	
	Household Income (AUD)	Affordability of Recommended Diet (%)	Household Income (AUD)	Affordability of Recommended Diet (%)	Household Income (AUD)	Affordability of Recommended Diet (%)	Household Income (AUD)	Affordability of Recommended Diet (%)
Median gross	3188.00	20.8	3248.18	21.2	3317.30	20.9	3377.40	23.2
Minimum wage disposable	2358.00	26.0	3336.02	19.3	2679.10	24.1	2765.86	26.4
Welfare dependent	1739.68	35.6	3084.52	20.3	1873.27	34.6	1937.15	37.7

* cost of diet divided by household income (‘affordable’ is <30% of income).

## Data Availability

The data presented in this study are available in this article and its Supplementary Materials.

## Supplementary Materials

The following supporting information can be downloaded at: https://www.mdpi.com/article/10.3390/ijerph20043146/s1, Figure S1: Total recommended diet and food group costs in all 10 locations (F to O) from 2019 to 2022; Figure S2: Total habitual diet and food group costs in 10 locations (F to O) from 2019 to 2022; Table S1: Total diet and food group costs by SEIFA quintile from 2019 to 2022; Table S2: Calculations of minimum wage disposable household incomes for the reference household per fortnight from 2019 to 2022; Table S3: Calculations of welfare-dependent household incomes for the reference household per fortnight from 2019 to 2022.
