# Supplementary material for: Healthy Food Prices Increased More Than the Prices of Unhealthy Options during the COVID-19 Pandemic and Concurrent Challenges to the Food System
Source: Int J Environ Res Public Health. 2023 Feb 10;20(4):3146. doi: 10.3390/ijerph20043146 (PMC9967271; doi:10.3390/ijerph20043146)
Supplement: Supplementary file 1 [file ijerph-20-03146-s001.zip › Supplementary Figure S1.pdf]

Supplementary Figure S1: Total recommended diet and food group costs in all 10 locations (F to O) from 2019 to 2022

Note: Locations F, G, and H were defined as SEIFA Quintile 1 (most disadvantaged) by the Australian Bureau of Statistics, locations I, J, K, and L were defined as SEIFA Quintile 3 (median disadvantaged), and locations M, N, and O were defined as SEIFA Quintile 5 (least disadvantaged).

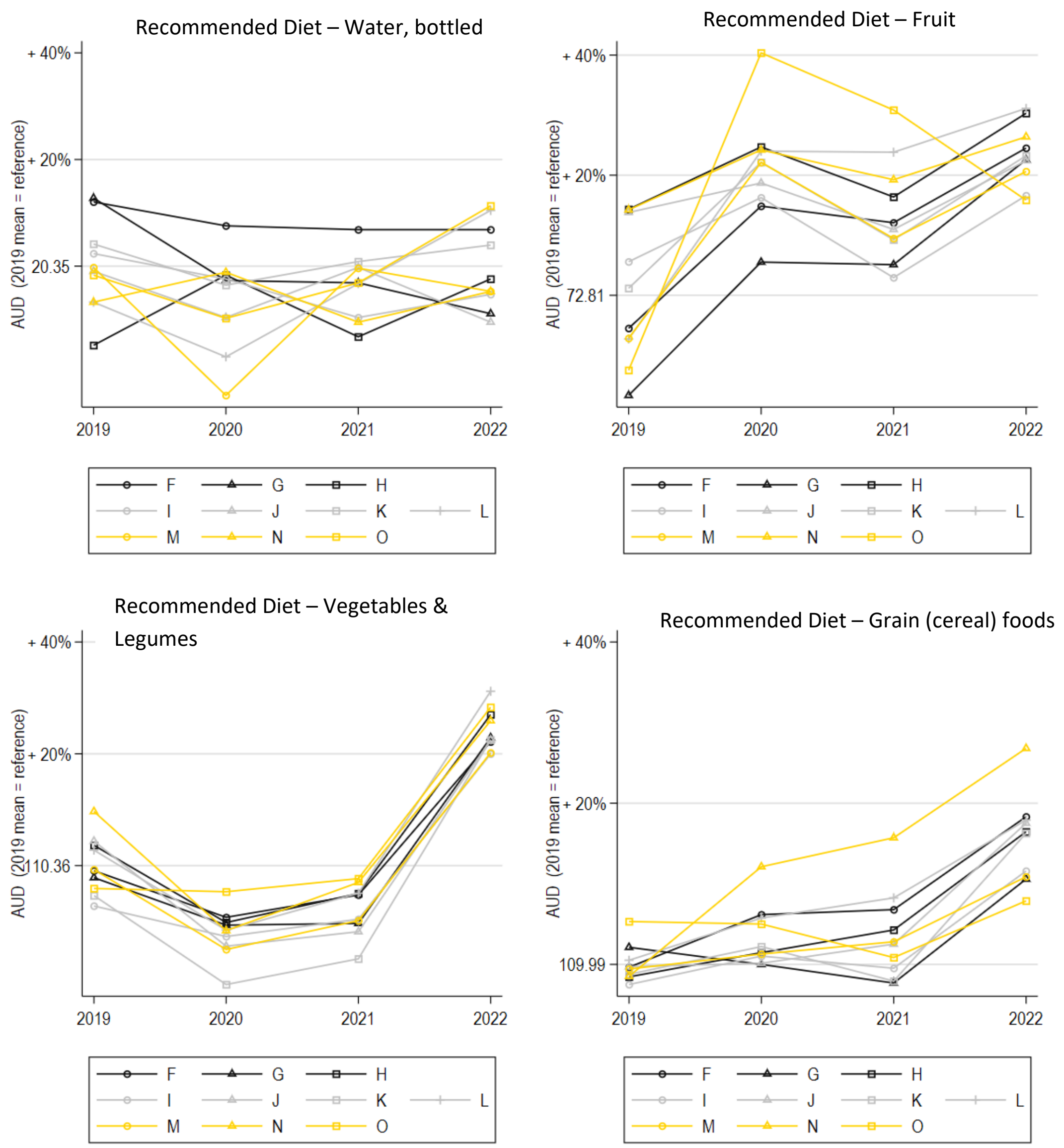

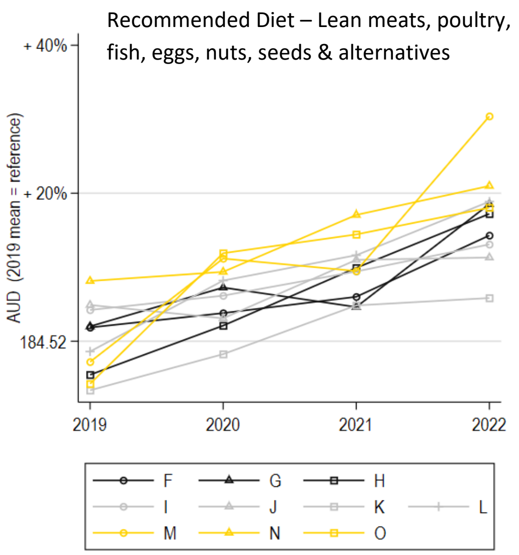
Recommended Diet – Lean meats, poultry, fish, eggs, nuts, seeds & alternatives
+ 40%
+ 20%
184.52
AUD (2019 mean = reference)
2019
2020
2021
2022
F
G
H
I
J
K
L
M
N
O

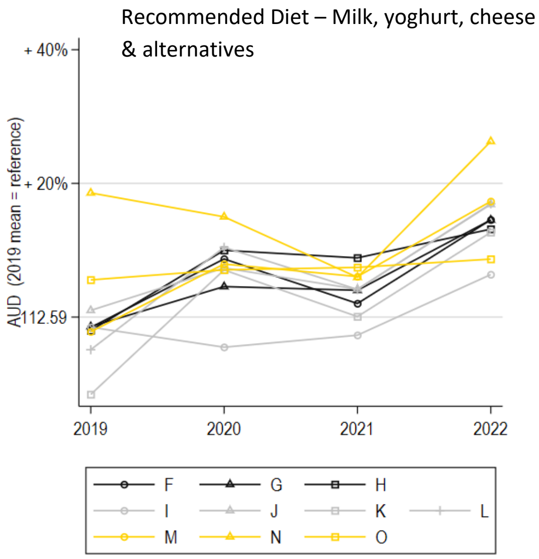
Recommended Diet – Milk, yoghurt, cheese & alternatives
+ 40%
+ 20%
112.59
AUD (2019 mean = reference)
2019
2020
2021
2022
F
G
H
I
J
K
L
M
N
O

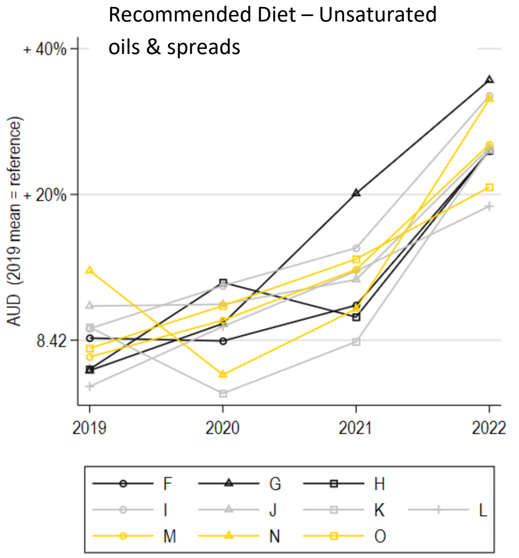
Recommended Diet – Unsaturated oils & spreads
+ 40%
+ 20%
8.42
AUD (2019 mean = reference)
2019
2020
2021
2022
F
G
H
I
J
K
L
M
N
O

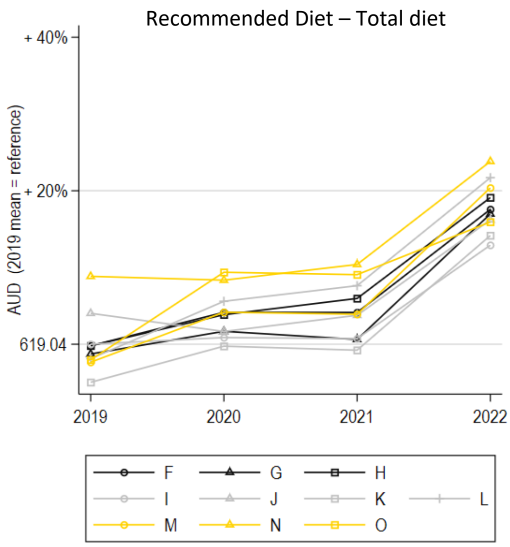
Recommended Diet – Total diet
+ 40%
+ 20%
619.04
AUD (2019 mean = reference)
2019
2020
2021
2022
F
G
H
I
J
K
L
M
N
O

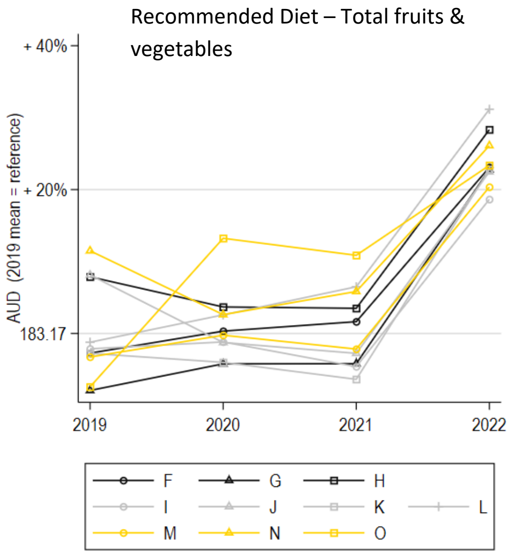
Recommended Diet – Total fruits & vegetables
+ 40%
+ 20%
183.17
AUD (2019 mean = reference)
2019
2020
2021
2022
F
G
H
I
J
K
L
M
N
O
