# Supplementary material for: Healthy Food Prices Increased More Than the Prices of Unhealthy Options during the COVID-19 Pandemic and Concurrent Challenges to the Food System
Source: Int J Environ Res Public Health. 2023 Feb 10;20(4):3146. doi: 10.3390/ijerph20043146 (PMC9967271; doi:10.3390/ijerph20043146)
Supplement: Supplementary file 1 [file ijerph-20-03146-s001.zip › Supplementary Figure S2.pdf]

Supplementary Figure S2: Total habitual diet and food group costs in 10 locations (F to O) from 2019 to 2022

Note: Locations F, G, and H were defined as SEIFA Quintile 1 (most disadvantaged) by the Australian Bureau of Statistics, locations I, J, K, and L were defined as SEIFA Quintile 3 (median disadvantaged), and locations M, N, and O were defined as SEIFA Quintile 5 (least disadvantaged).

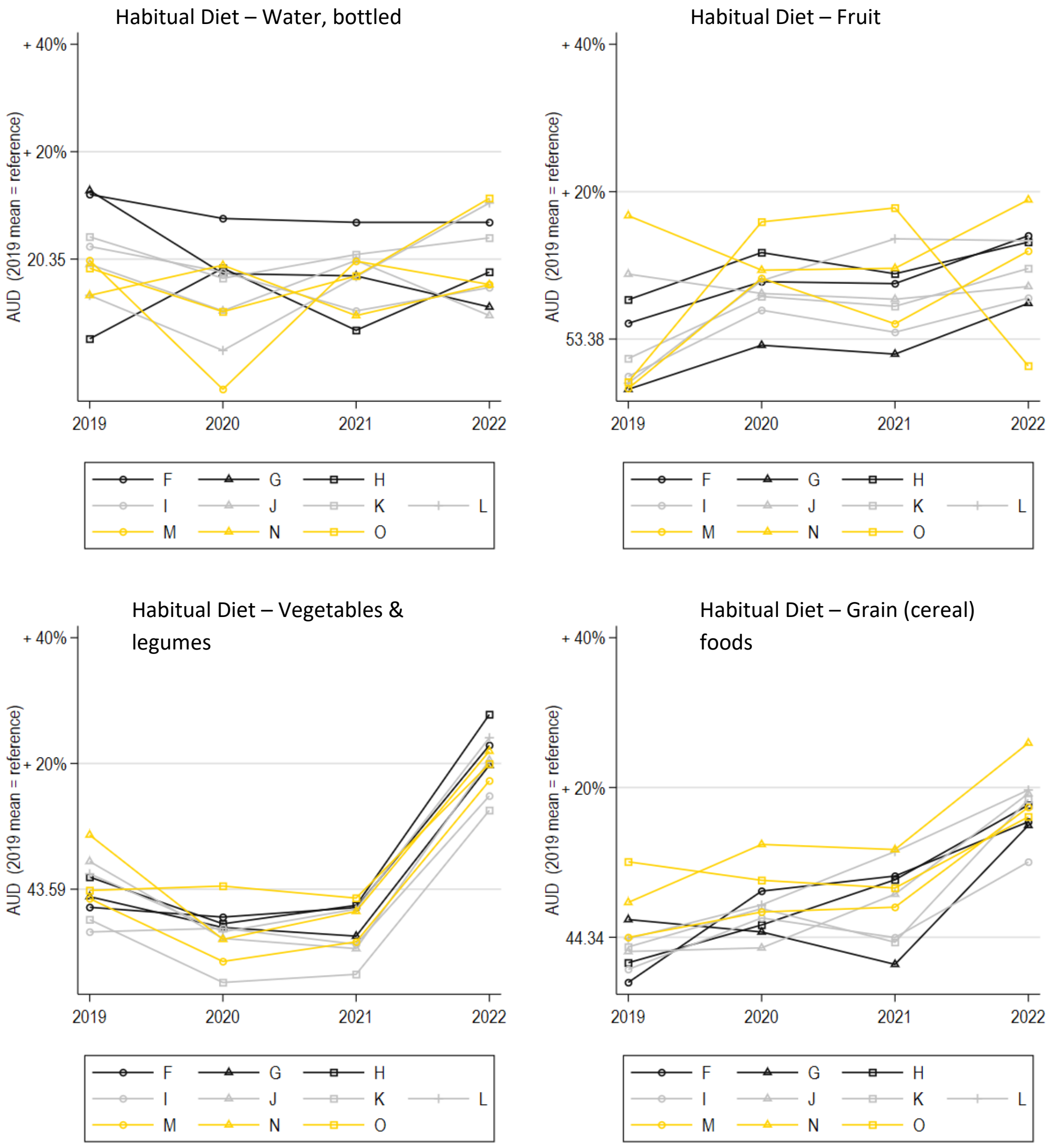

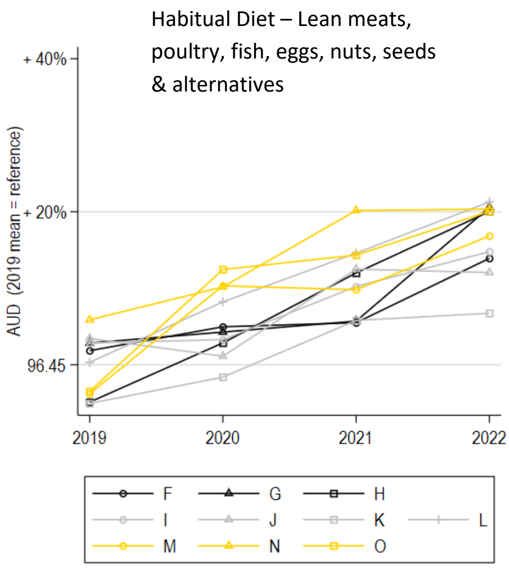
Habitual Diet – Lean meats, poultry, fish, eggs, nuts, seeds & alternatives
+ 40%
+ 20%
96.45
AUD (2019 mean = reference)
2019
2020
2021
2022
F
G
H
I
J
K
L
M
N
O

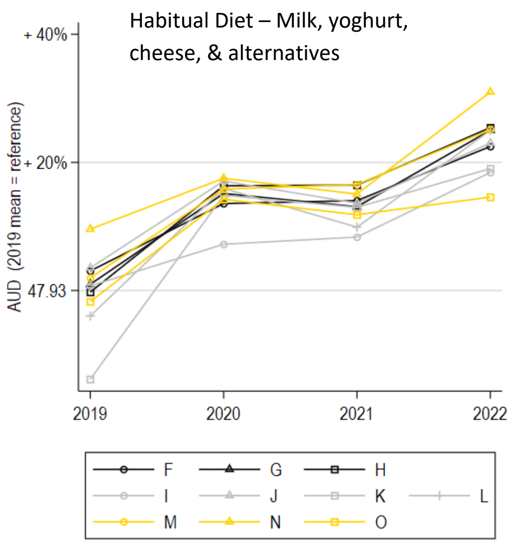
Habitual Diet – Milk, yoghurt, cheese, & alternatives
+ 40%
+ 20%
47.93
AUD (2019 mean = reference)
2019
2020
2021
2022
F
G
H
I
J
K
L
M
N
O

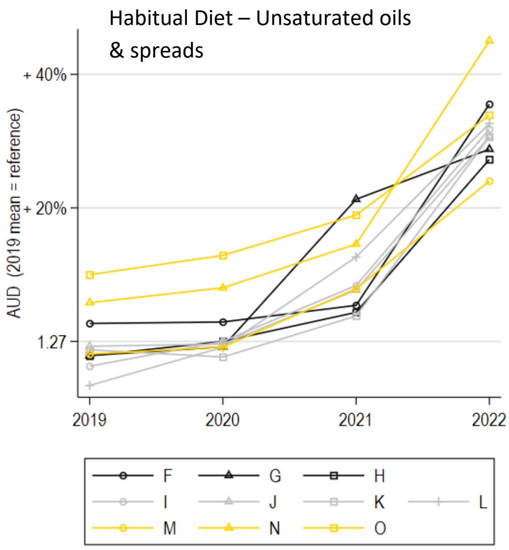
Habitual Diet – Unsaturated oils & spreads
+ 40%
+ 20%
1.27
AUD (2019 mean = reference)
2019
2020
2021
2022
F
G
H
I
J
K
L
M
N
O

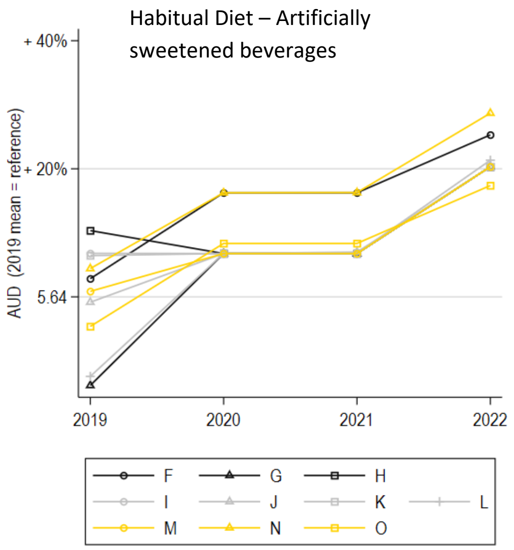
Habitual Diet – Artificially sweetened beverages
+ 40%
+ 20%
5.64
AUD (2019 mean = reference)
2019
2020
2021
2022
F
G
H
I
J
K
L
M
N
O

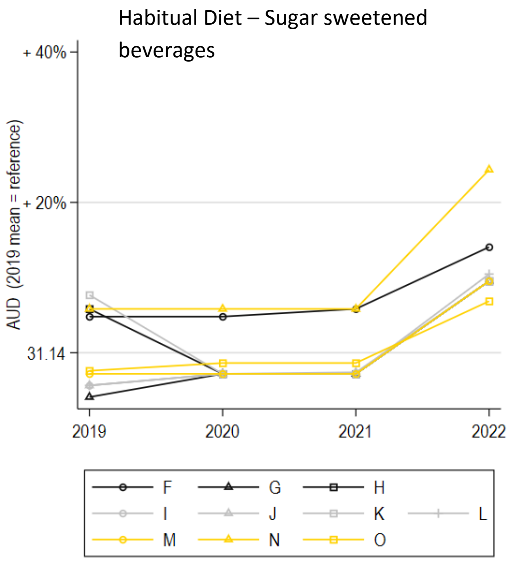
Habitual Diet – Sugar sweetened beverages
+ 40%
+ 20%
31.14
AUD (2019 mean = reference)
2019
2020
2021
2022
F
G
H
I
J
K
L
M
N
O

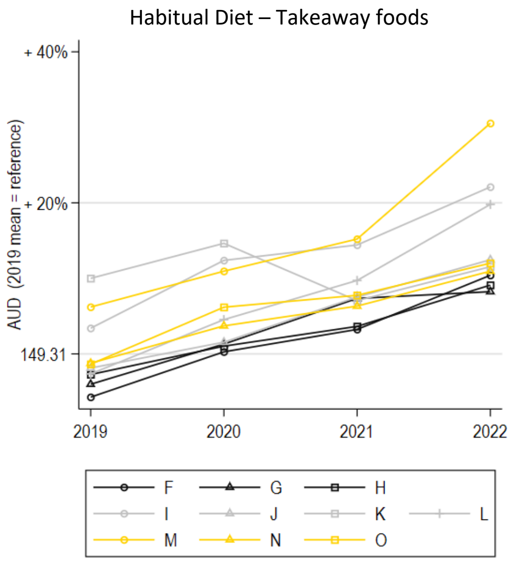
Habitual Diet – Takeaway foods
+ 40%
+ 20%
149.31
AUD (2019 mean = reference)
2019
2020
2021
2022
F
G
H
I
J
K
L
M
N
O

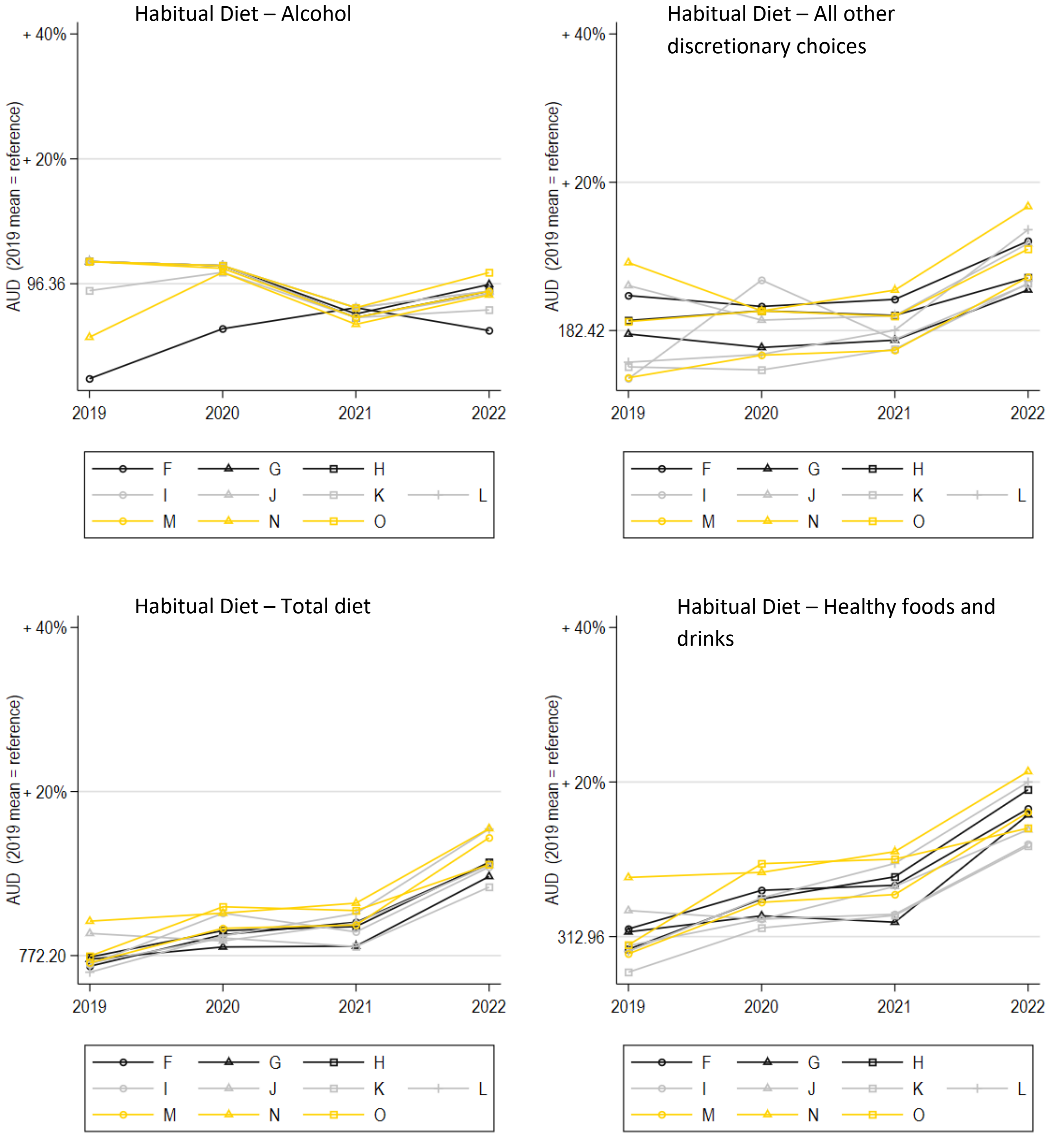
Habitual Diet – Alcohol
+ 40%
+ 20%
96.36
AUD (2019 mean = reference)
2019
2020
2021
2022
F
G
H
I
J
K
L
M
N
O
Habitual Diet – All other discretionary choices
+ 40%
+ 20%
182.42
AUD (2019 mean = reference)
2019
2020
2021
2022
F
G
H
I
J
K
L
M
N
O
Habitual Diet – Total diet
+ 40%
+ 20%
772.20
AUD (2019 mean = reference)
2019
2020
2021
2022
F
G
H
I
J
K
L
M
N
O
Habitual Diet – Healthy foods and drinks
+ 40%
+ 20%
312.96
AUD (2019 mean = reference)
2019
2020
2021
2022
F
G
H
I
J
K
L
M
N
O

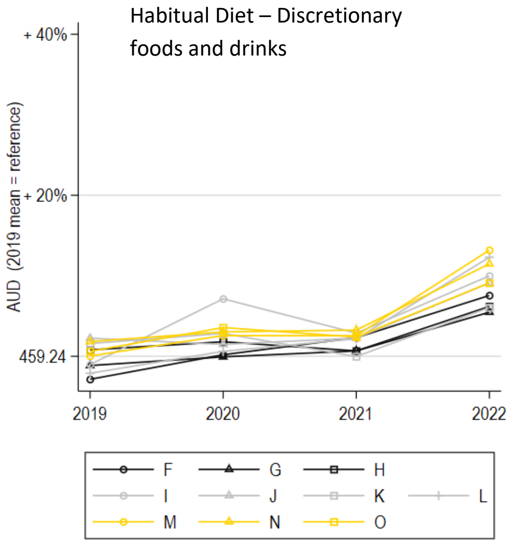
Habitual Diet – Discretionary foods and drinks
AUD (2019 mean = reference)
+ 40%
+ 20%
459.24
2019
2020
2021
2022
F
G
H
I
J
K
L
M
N
O

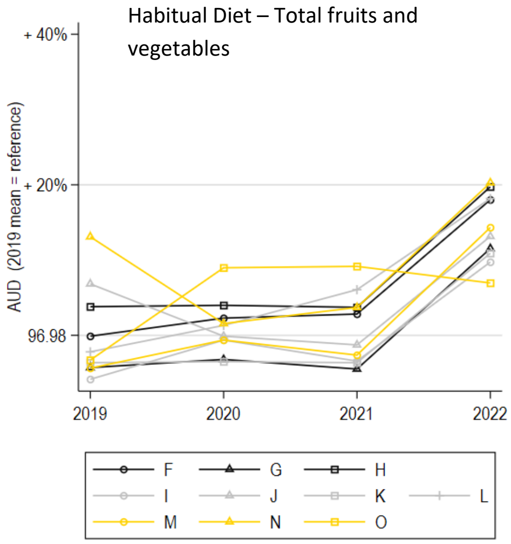
Habitual Diet – Total fruits and vegetables
AUD (2019 mean = reference)
+ 40%
+ 20%
96.98
2019
2020
2021
2022
F
G
H
I
J
K
L
M
N
O

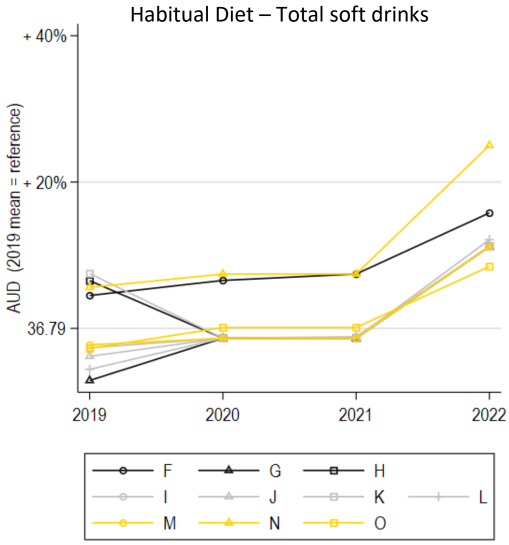
Habitual Diet – Total soft drinks
AUD (2019 mean = reference)
+ 40%
+ 20%
36.79
2019
2020
2021
2022
F
G
H
I
J
K
L
M
N
O
