# Supplementary material for: Healthy Food Prices Increased More Than the Prices of Unhealthy Options during the COVID-19 Pandemic and Concurrent Challenges to the Food System
Source: Int J Environ Res Public Health. 2023 Feb 10;20(4):3146. doi: 10.3390/ijerph20043146 (PMC9967271; doi:10.3390/ijerph20043146)
Supplement: Supplementary file 1 [file ijerph-20-03146-s001.zip › Supplementary Table S1.pdf]

Supplementary Table S1: Total diet and food group costs of the habitual diet for the reference household

	**Total diet and food group costs of the habitual diet for the reference household**														
**Income Quintile**	**Q1 – most disadvantaged**					**Q3 – median disadvantage**					**Q5 – least disadvantaged**				
**Year**	**2019**	**2020**	**2021**	**2022**	**% Change 2019-2022**	**2019**	**2020**	**2021**	**2022**	**% Change 2019-2022**	**2019**	**2020**	**2021**	**2022**	**% Change 2019-2022**
**Food/food groups**	**Mean cost (A$) ± SE**	**Mean cost (A$) ± SE**	**Mean cost (A$) ± SE**	**Mean cost (A$) ± SE**		**Mean cost (A$) ± SE**	**Mean cost (A$) ± SE**	**Mean cost (A$) ± SE**	**Mean cost (A$) ± SE**		**Mean cost (A$) ± SE**	**Mean cost (A$) ± SE**	**Mean cost (A$) ± SE**	**Mean cost (A$) ± SE**	
Water, bottled	21.03 ± 1.51	20.57 ± 0.54	19.70 ± 0.96	20.05 ± 0.76	-4.7%	20.29 ± 0.42	18.69 ± 0.59	19.72 ± 0.42	20.28 ± 0.83	0%	19.76 ± 0.33	17.97 ± 1.12	19.40 ± 0.50	20.48 ± 0.89	+3.6%
Fruit	53.50 ± 1.59	56.71 ± 1.62	55.94 ± 1.50	59.08 ± 1.27	+10.4%	52.73 ± 1.58	56.56 ± 0.38	56.64 ± 1.24	58.14 ± 0.78	+10.2%	54.13 ± 3.34	59.34 ± 1.04	58.63 ± 1.98	58.22 ± 2.90	+7.6%
Vegetables (& legumes)	43.28 ± 0.50	41.26 ± 0.17	41.71 ± 0.56	53.82 ± 0.84	+24.4%	43.07 ± 1.03	39.71 ± 0.76	39.79 ± 0.81	51.44 ± 0.99	+19.4%	44.60 ± 1.13	40.84 ± 1.27	41.66 ± 0.73	52.19 ± 0.49	+17.0%
Grain (cereal) foods	43.29 ± 0.90	45.60 ± 0.61	46.15 ± 1.39	51.44 ± 0.30	+18.8%	43.49 ± 0.33	45.37 ± 0.50	46.17 ± 1.09	51.8 ± 0.87	+19.1%	46.51 ± 1.06	47.79 ± 0.94	47.63 ± 0.82	53.11 ± 1.12	+14.2%
Lean meats, poultry, fish, eggs, nuts, seeds & alternatives	96.39 ± 1.91	100.34 ± 0.49	103.91 ± 1.68	113.97 ± 1.68	+18.2%	96.82 ± 1.62	99.11 ± 1.74	106.85 ± 1.57	109.67 ± 2.53	+13.3%	96.01 ± 2.50	107.04 ± 0.58	110.70 ± 2.36	114.84 ± 0.89	+19.6%
Milk, yoghurt, cheese & alternatives	48.53 ± 0.38	55.13 ± 0.31	54.89 ± 0.40	59.58 ± 0.36	+22.8%	46.31 ± 1.58	54.54 ± 0.93	53.31 ± 0.52	58.18 ± 0.66	+25.6%	49.5 ± 1.30	55.54 ± 0.37	54.87 ± 0.54	59.23 ± 1.88	+19.6%
Unsaturated oils and spreads	1.27 ± 0.02	1.28 ± 0.01	1.41 ± 0.06	1.66 ± 0.03	+31.2%	1.23 ± 0.01	1.26 ± 0.01	1.38 ± 0.02	1.67 ± 0.01	+35.6%	1.33 ± 0.04	1.36 ± 0.04	1.45 ± 0.03	1.71 ± 0.06	+28.4%
Artificially sweetened beverages	5.63 ± 0.33	6.20 ± 0.15	6.20 ± 0.15	6.88 ± 0.08	+22.2%	5.64 ± 0.22	6.02 ± 0.00	6.03 ± 0.00	6.80 ± 0.01	+20.3%	5.65 ± 0.12	6.23 ± 0.14	6.23 ± 0.14	6.89 ± 0.16	+21.9%
Sugar sweetened beverages	31.64 ± 0.95	31.06 ± 0.65	31.17 ± 0.73	34.58 ± 0.38	+6.2%	30.73 ± 0.81	30.27 ± 0.00	30.29 ± 0.01	34.19 ± 0.06	+12.2%	31.21 ± 0.72	31.32 ± 0.68	31.32 ± 0.68	35.37 ± 1.38	+10.2%
Takeaway foods	143.10 ± 1.06	150.61 ± 0.37	156.4 ± 1.59	163.13 ± 0.77	-0.2%	152.63 ± 3.78	161.67 ± 4.03	163.77 ± 2.18	173.92 ± 3.40	-4.3%	151.11 ± 3.05	159.69 ± 2.59	163.89 ± 3.36	175.94 ± 7.75	+0.1%
Alcoholic beverages	93.76 ± 4.92	95.91 ± 2.65	91.84 ± 0.36	93.54 ± 1.81	+14.0%	98.66 ± 0.97	98.89 ± 0.22	91.91 ± 0.38	94.4 ± 0.60	+13.9%	95.90 ± 3.18	98.67 ± 0.25	91.31 ± 0.61	95.98 ± 0.87	+16.4%
All other discretionary choices	185.82 ± 2.26	184.63 ± 2.61	185.43 ± 2.38	197.43 ± 2.94	+9.3%	178.04 ± 4.52	182.27 ± 4.25	181.66 ± 1.51	199.74 ± 2.96	+11.3%	184.86 ± 6.68	183.62 ± 2.96	185.29 ± 3.50	203.66 ± 4.12	+13.3%
**Total diet**	**767.25 ± 2.11**	**789.30 ± 3.78**	**794.74 ± 5.65**	**855.17 ± 3.47**	**+11.5%**	**769.64 ± 6.96**	**794.37 ± 5.18**	**797.49 ± 5.74**	**860.23 ± 9.84**	**+11.8%**	**780.57 ± 9.92**	**809.40 ± 4.91**	**812.38 ± 4.93**	**877.63 ± 8.28**	**+12.4%**
Healthy foods and drinks	312.92 ± 2.09	327.09 ± 2.47	329.90 ± 4.61	366.49 ± 2.50	+17.1%	309.59 ± 4.49	321.26 ± 2.24	329.87 ± 4.36	357.98 ± 5.24	+15.6%	317.50 ± 7.98	336.11 ± 3.88	340.57 ± 4.38	366.67 ± 5.58	+15.5%
Discretionary foods and drinks	454.33 ± 3.95	462.20 ± 2.20	464.83 ± 2.06	488.68 ± 2.28	+7.6%	460.06 ± 4.13	473.11 ± 5.76	467.62 ± 2.53	502.25 ± 5.14	+9.2%	463.07 ± 1.98	473.29 ± 1.09	471.8 ± 0.99	510.96 ± 4.44	+10.3%

	Total diet and food group costs of the recommended diet for the reference household														
**Income Quintile**	**Q1**					**Q3**					**Q5**				
**Year**	**2019**	**2020**	**2021**	**2022**	**% Change 2019-2022**	**2019**	**2020**	**2021**	**2022**	**% Change 2019-2022**	**2019**	**2020**	**2021**	**2022**	**% Change 2019-2022**
**Food/food groups**	**Mean cost (A$) ± SE**	**Mean cost (A$) ± SE**	**Mean cost (A$) ± SE**	**Mean cost (A$) ± SE**		**Mean cost (A$) ± SE**	**Mean cost (A$) ± SE**	**Mean cost (A$) ± SE**	**Mean cost (A$) ± SE**		**Mean cost (A$) ± SE**	**Mean cost (A$) ± SE**	**Mean cost (A$) ± SE**	**Mean cost (A$) ± SE**	
Water, bottled	21.03 ± 1.51	20.57 ± 0.54	19.70 ± 0.96	20.05 ± 0.76	-4.7%	20.29 ± 0.42	18.69 ± 0.59	19.72 ± 0.42	20.28 ± 0.83	0%	19.76 ± 0.33	17.97 ± 1.12	19.40 ± 0.50	20.48 ± 0.89	+3.6%
Fruit	70.90 ± 5.39	83.75 ± 3.29	80.95 ± 1.95	91.60 ± 1.38	+29.2%	75.23 ± 2.77	87.57 ± 1.09	81.36 ± 2.76	89.81 ± 1.88	+19.4%	71.49 ± 4.85	93.86 ± 3.43	87.27 ± 3.67	88.06 ± 1.81	+23.2%
Vegetables (& legumes)	110.57 ± 1.58	99.29 ± 0.37	102.79 ± 1.57	136.87 ± 1.37	+23.8%	108.87 ± 2.77	93.83 ± 2.08	98.49 ± 2.33	136.89 ± 2.35	+25.7%	112.16 ± 3.73	98.84 ± 2.74	104.78 ± 2.16	137.75 ± 2.18	+22.8%
Grain (cereal) foods	110.07 ± 0.97	112.78 ± 1.67	113.20 ± 2.44	126.62 ± 2.09	+15%	109.03 ± 0.62	112.50 ± 1.17	112.25 ± 2.17	127.39 ± 1.39	+16.8%	111.19 ± 1.91	116.74 ± 2.85	117.09 ± 4.19	126.69 ± 5.28	+13.9%
Lean meats, poultry, fish, eggs, nuts, seeds & alternatives	184.10 ± 3.25	192.62 ± 2.28	197.15 ± 2.39	215.3 ± 1.89	+16.9%	185.02 ± 4.31	191.76 ± 3.46	201.53 ± 2.45	207.17 ± 4.29	+12.0%	184.26 ± 6.37	204.47 ± 1.13	209.73 ± 3.35	227.24 ± 5.62	+23.3%
Milk, yoghurt, cheese & alternatives	110.73 ± 0.16	121.32 ± 1.5	118.18 ± 1.86	128.45 ± 0.42	+16.0%	107.82 ± 2.66	118.31 ± 3.20	114.19 ± 1.64	127.47 ± 2.43	+18.2%	120.83 ± 5.57	123.85 ± 2.31	119.89 ± 0.44	132.21 ± 4.67	+9.4%
Unsaturated oils and spreads	8.20 ± 0.10	8.70 ± 0.16	9.21 ± 0.37	10.88 ± 0.22	+32.7%	8.46 ± 0.17	8.57 ± 0.24	9.06 ± 0.20	10.62 ± 0.23	+25.6%	8.59 ± 0.26	8.50 ± 0.20	9.12 ± 0.14	10.69 ± 0.24	+24.4%
**Total diet**	**615.60 ± 1.79**	**639.03 ± 3.93**	**641.18 ± 7.93**	**729.76 ± 3.21**	**+18.5%**	**614.70 ± 10.08**	**631.23 ± 6.81**	**636.60 ± 9.95**	**719.63 ± 10.40**	**+17.1%**	**628.29 ± 18.60**	**664.22 ± 8.05**	**667.27 ± 10.01**	**743.11 ± 11.54**	**+18.3%**
