# Supplementary material for: Healthy Food Prices Increased More Than the Prices of Unhealthy Options during the COVID-19 Pandemic and Concurrent Challenges to the Food System
Source: Int J Environ Res Public Health. 2023 Feb 10;20(4):3146. doi: 10.3390/ijerph20043146 (PMC9967271; doi:10.3390/ijerph20043146)
Supplement: Supplementary file 1 [file ijerph-20-03146-s001.zip › Supplementary Tables S2 and S3.pdf]

Supplementary Table S2: Calculations of minimum wage disposable household incomes for the reference household per fortnight

| | Reference household: Two parents with two children (Adult male (between 31 and 50 years), adult female (between 31 and 50 years), 14 years old boy, 8 years old girl) | | | | | | | |
|---|---|---|---|---|---|---|---|---|
| **Assumptions** | • The adult male works on a permanent basis for 38 hours/week, the adult female works on a part-time basis for 6 hours/week, both at national minimum wage rates ($19.49/hr in 2019 & 2020, $20.33/hr in 2021)<br>• Both children attend school and are fully immunised. None of the family have a disability.<br>• The family has some emergency savings that earn negligible interest, and is privately renting a 3 bedroom house at $376/week | | | | | | | |
| | **Aug 2019** | | **May 2020** | | **Sep 2021** | | **Sep 2022** | |
| **Income** | **Amount** | **Amount/ fortnight** | **Amount** | **Amount/ fortnight** | **Amount** | **Amount/ fortnight** | **Amount** | **Amount/ fortnight** |
| Paid employment - adult male | $19.49/hr for 38h/week | $1,481.24 | $19.49/hr for 38h/week | $1,481.24 | $20.33/hr for 38h/week | $1,545.08 | $21.38/hr for 38h/week | $1,624.88 |
| Paid employment - adult female | $19.49/hr for 6h/week | $233.88 | $19.49/hr for 6h/week | $233.88 | $20.33/hr for 6h/week | $243.96 | $21.38/hr for 6h/week | $256.56 |
| JobSeeker Allowance - adult female | N/A | $ - | $324.05/fortnight | $324.05 | $273.70/fortnight | $273.70 | $258.80/fortnight | $258.80 |
| Family Tax Benefit A fortnightly payment | $428.40/fortnight | $428.40 | $428.40/fortnight | $428.40 | $440.02/fortnight | $440.02 | $455.42/fortnight | $455.42 |
| Family Tax Benefit A annual supplement | $751.90/child/year | $57.84 | $766.50/child/year | $58.96 | $788.40/child/year | $60.65 | $817.60/child/year | $62.89 |
| Family Tax Benefit B fortnightly payment | $107.66/fortnight | $107.66 | $74.06/fortnight | $74.06 | $55.16/fortnight | $55.16 | $61.18/fortnight | $61.68 |
| Family Tax Benefit B annual supplement | $365.00/year/family | $14.04 | $372.30/year/family | $14.32 | $383.25/year/family | $14.74 | $397.85/year/family | $15.30 |
| Total Clean Energy Supplement (from all payments) | included in Family Tax Benefit estimator amounts | $ - | included in Family Tax Benefit estimator amounts +$7.90 | $7.90 | included in FTB/JobSeeker estimator amounts | $ - | included in FTB/JobSeeker estimator amounts | $ - |
| Rent Assistance | $161.14/fortnight | $161.14 | $164.08/fortnight | $164.08 | $165.62/fortnight | $165.62 | $171.50/fortnight | $171.50 |
| First Economic Support payment | N/A | $ - | $750 paid in April 2020 (assumed spent over 6 fortnights) | $125.00 | N/A | $ - | N/A | $ - |
| Coronavirus Supplement | N/A | $ - | $550/fortnight from April 27 | $550.00 | N/A | $ - | N/A | $ - |
| Income Tax Paid (fortnightly) | Tax p.a. less low & low-middle income tax offsets | ($125.87) | Tax p.a. less low & low-middle income tax offsets (nil payable) | $ - | Tax p.a. less low & low-middle income tax offsets | ($119.82) | Tax p.a. less low & low-middle income tax offsets | ($141.17) |
| **Fortnightly income total** | | **$2,358.33** | | **$3,336.02** | | **$2,679.10** | | **$2,765.86** |

Supplementary Table S3: Calculations of welfare dependent household incomes for the reference household per fortnight

| | | | | | | | | |
|---|---|---|---|---|---|---|---|---|
| | **Reference household: Two parents with two children (**Adult male (between 31 and 50 years), adult female (between 31 and 50 years), 14 years old boy, 8 years old girl) | | | | | | | |
| **Assumptions** | • The adult male works on a permanent basis for 38 hours/week, the adult female works on a part-time basis for 6 hours/week, both at national minimum wage rates ($19.49/hr in 2019 & 2020, $20.33/hr in 2021)<br>• Both children attend school and are fully immunised. None of the family have a disability.<br>• The family has some emergency savings that earn negligible interest, and is privately renting a 3 bedroom house at $376/week | | | | | | | |
| | **Aug 2019** | | **May 2020** | | **Sep 2021** | | **Sep 2022** | |
| **Income** | **Amount** | **Amount/ fortnight** | **Amount** | **Amount/ fortnight** | **Amount** | **Amount/ fortnight** | **Amount** | **Amount/ fortnight** |
| Newstart Allowance - adult male | $504.7/fortnight | $504.70 | $510.80/fortnight | $510.80 | $565.40/fortnight | $565.40 | $593.20/fortnight | $593.20 |
| Newstart Allowance - adult female | $504.7/fortnight | $504.70 | $510.80/fortnight | $510.80 | $565.40/fortnight | $565.40 | $593.20/fortnight | $593.20 |
| Family Tax Benefit A fortnightly payment | $428.40/fortnight | $428.40 | $428.40/fortnight | $428.40 | $440.02/fortnight | $440.02 | $455.42/fortnight | $455.42 |
| Family Tax Benefit A annual supplement | $751.90/child/year | $57.84 | $766.50/child/year | $58.96 | $788.40/child/year | $60.65 | $817.60/child/year | $62.89 |
| Family Tax Benefit B fortnightly payment | $52.08/fortnight | $52.08 | $31.36/fortnight | $31.36 | $45.64/fortnight | $45.64 | $45.64/fortnight | $45.64 |
| Family Tax Benefit B annual supplement | $365.00/year/family | $14.04 | $372.30/year/family | $ 14.32 | $383.25/year/family | $14.74 | $397.85/year/family | $15.30 |
| Total Clean Energy Supplement (from all payments) | $7.90 each | $15.80 | $7.90 each | $15.80 | $7.90 each | $15.80 | included in FTB/JobSeeker estimator amounts | $ - |
| Rent Assistance | $162.12 | $162.12 | $164.08 | $164.08 | $165.62/fortnight | $165.62 | $171.50/fortnight | $171.50 |
| Economic Support payment | N/A | $ - | 2x$750 in 2020 | $250.00 | N/A | $ - | N/A | $ - |
| Coronavirus Supplement | N/A | $ - | 2x $550/fortnight from 27 April | $1,100.00 | N/A | $ - | N/A | $ - |
| Income Tax Paid (fortnightly) | Nil payable | $ - | Nil payable | $ - | Nil payable | $ - | Nil payable | $ - |
| **Fortnightly income total** | | **$1,739.68** | | **$3,084.52** | | **$1,873.27** | | **$1,937.15** |
